# Electrochemical Exfoliation of Layered Non‐van der Waals Crystals into 2D Nanosheets: MAX Phases and Beyond

**DOI:** 10.1002/smll.202408801

**Published:** 2025-02-09

**Authors:** Lei Zheng, Heng Li, Evgeniya Kovalska, Jan Luxa, Ruizhi Yu, Huaijuan Zhou, Manfang Chen, Bing Wu, Zdenek Sofer

**Affiliations:** ^1^ Department of Inorganic Chemistry University of Chemistry and Technology Prague Technická 5 Prague 166 28 Czech Republic; ^2^ Department of Engineering Faculty of Environment Science and Economy University of Exeter Exeter EX4 4QF UK; ^3^ Institute of Micro/Nano Materials and Devices Ningbo University of Technology Ningbo Zhejiang 315211 P. R. China; ^4^ Advanced Research Institute of Multidisciplinary Sciences Beijing Institute of Technology Beijing 100081 China; ^5^ National Base for International Science & Technology Cooperation School of Chemistry Xiangtan University Xiangtan 411105 China

**Keywords:** 2D materials, delocalization, electrochemical exfoliation, layered non‐van der Waals

## Abstract

2D materials have rapidly gained attention due to their exceptional properties like high surface area, flexibility, and tunable electronic characteristics. These attributes make them highly versatile for applications in energy storage, electronics, and biomedicine. Inspired by graphene’s success, researchers are exploring other 2D materials from bulk crystals. Electrochemical exfoliation (ECE) is an efficient method for producing these materials, offering more sustainable mild conditions, quick processing, simple equipment, and high yields. While substantial progress has been made in the ECE of layered van der Waals (L‐vdW) crystals, the exploration of layered non‐van der Waals (L‐NvdW) materials remains in its early stages. This review delves into using ECE to create 2D nanoplatelets from L‐NvdW crystals. A comparative analysis of exfoliation techniques is provided for L‐vdW and L‐NvdW materials, followed by a comprehensive overview of recent advances in ECE methods applied to L‐NvdW crystals. The discussion is organized around key categories, including the selective extraction of “M” and “A” layers respectively from MAX phases, decalcification of Zintl phases, and oxide delocalization from metal oxides. It is concluded by highlighting the potential applications of these 2D materials and discussing the challenges and future directions in this evolving field.

## Graphical abstract

This review offers the first comprehensive analysis of electrochemical exfoliation (ECE) for layered non‐van der Waals (L‐NvdW) materials, compares exfoliation methods with L‐vdW materials, and introduces novel techniques for selective layer extraction in MAX phases, Zintl phases CaSi*_x_* Ge*_y_*, and metal oxides, highlighting both the potential and challenges of L‐NvdW materials for future advancements.

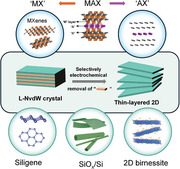

## 1 Introduction

2D materials have attracted significant attention due to their unique characteristics, such as high surface area, mechanical flexibility, and tunable electronic properties and structures.^[^ ^1^ ^]^ These properties make them widely applicable in various fields such as energy storage and conversions, flexible devices, optoelectronics, and biomedicines.^[^ ^2^, ^3^ ^]^ The surge of interest in graphene research has sparked global enthusiasm for exfoliation and developing other 2D families, which exhibit a vast array of chemical structures.^[^ ^4^ ^]^ These materials include insulatores (e.g., hBN and PbS),^[^ ^5^ ^]^ monoelemental semiconductors/semi‐metals (e.g., phosphorene, arsenene, tellurene, bismuthene, antimonene),^[^ ^6^, ^7^ ^]^ transition metal dichalcogenides (TMDs, e.g., 1T/2H/3R‐MoS_2_, WS_2_, MoSe_2_, MoTe_2_, NbS_2_),^[^ ^8^ ^]^ metal phosphorous trichalcogenides (MPCh_3_, e.g., Fe/Co/Ni/Mn/Zn/CoPS_3_, Cu_0.5_In_0.5_PS_3_, Ag_0.5_In_0.5_PS_3_, MgPSe_3_),^[^ ^9^, ^10^ ^]^ transition metal oxides (TMOs, e.g., MoO_3_, V_2_O_5_, TiO_2_),^[^ ^11^ ^]^ MXenes,^[^ ^12^ ^]^ and layered double hydroxides (LDHs).^[^ ^13^ ^]^ The electron confinement in ultrathin 2D anisotropy endows these materials with compelling electronic characteristics, ranging from insulators and metals to spin‐active systems, often exhibiting unique properties not found in their 3D counterparts, making them promising candidates for fundamental condensed matter investigations and microelectronic applications.^[^ ^14^, ^15^ ^]^ Moreover, utilizing 2D materials as foundational elements enables the creation of diverse van der Waals (vdW) heterostructures that display novel properties not present in the individual components. These heterostructures can include combinations such as 2D/2D, 0D/2D, 1D/2D, and 3D/2D configurations, offering unprecedented functionalities and broadening the scope of material innovation.^[^ ^16^, ^17^ ^]^ Interestingly, stacking two graphene layers with a twisted angle creates a moiré pattern, leading to new electronic states and remarkable physical phenomena, such as flat bands that enhance electron correlation effects, resulting in unconventional superconductivity and other quantum phenomena.^[^ ^18^, ^19^, ^20^ ^]^ This approach has been applied to other 2D materials, such as MoS_2_, extending the exploration of these unique properties beyond graphene.^[^ ^21^, ^22^, ^23^ ^]^

**Figure 1:**
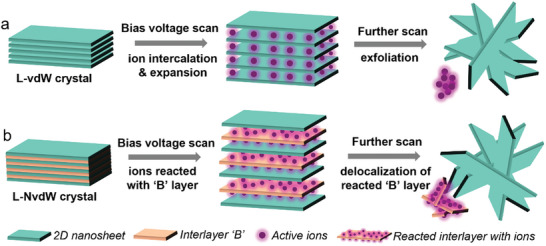
Comparison of electrochemical exfoliation for a) layered van der Waals crystal and b) layered non‐van der Waals crystal.

The preparation of 2D materials generally follows two approaches: bottom‐up and top‐down.^[^ ^24^, ^25^, ^26^ ^]^ Bottom‐up methods, such as chemical vapor deposition (CVD) and molecular beam epitaxy (MBE), involve assembling materials from atomic or molecular precursors. These methods allow precise control over the material's thickness, composition, and properties, enabling the production of high‐quality, large‐area films. However, they often require complex equipment, and high temperatures, and can be expensive and time‐consuming. On the other hand, top‐down methods, such as mechanical exfoliation, liquid‐phase exfoliation, and electrochemical exfoliation, involve breaking down high‐quality bulk crystals into thin layers. These methods utilize weak vdW forces, typically ranging from 30 to 60 meV, to hold adjacent layers together. In contrast, strong covalent bonding, usually around 7.3 eV, is found within the layers.^[^ ^27^ ^]^ Techniques that can disrupt these forces while preserving the strong covalent intralayer interactions are particularly effective. However, layered non‐van der Waals (L‐NvdW) materials exhibit strong covalent bonding both between and within layers. Since there is no van der Waals gap to facilitate easy cleavage, the exfoliation of these compounds necessitates the breaking of chemical bonds. Among these, electrochemical exfoliation (ECE) is especially notable for its efficiency, scalability, and ability to produce high‐quality 2D materials with controlled thickness.^[^ ^28^, ^29^, ^30^ ^]^ Typically, as schematically depicted in **Figure** **1**a, this method employs an electric field under bias voltage to intercalate ions between layers, weakening the vdW forces and facilitating the separation of layers, providing a rapid and environmentally friendly alternative to conventional exfoliation techniques. The 2D thin layers obtained through this approach maintain the same chemical composition as the bulk parent crystal. Additionally, electrochemical exfoliation can not only delaminate layered van der Waals‐type (L‐vdW) 2D crystals but also perform electrochemical etching and exfoliation on L‐NvdW materials.^[^ ^31^, ^32^ ^]^ As shown in Figure 1b, by selectively delocalizing components in specific layers while other groups terminate the remaining layers, leading to an expansion of the interlayer spacing and partial exfoliation, and with subsequent ultrasonic treatment, thin‐layer 2D materials can be produced. This method is fundamentally different from the liquid‐phase exfoliation of NvdW crystals.^[^ ^33^, ^34^ ^]^ Particularly, the resulting 2D nanoplates differ chemically from their parent bulk crystals.

The ECE of L‐vdW materials to produce thin 2D layers has been extensively studied, with several comprehensive reviews available on this topic.^[^ ^5^, ^35^, ^36^, ^37^, ^38^ ^]^ However, research on the electrochemical exfoliation of L‐NvdW materials is still in its infancy, with only a few studies reported and no reviews published to date. Our goal is to provide an overview and perspective on the use of the ECE process for L‐NvdW 3D‐bonded crystals to produce 2D nanoplatelets. This manuscript includes a comparison of thin‐layer 2D materials prepared using ECE from L‐vdW and L‐NvdW crystals, a brief introduction to ECE, followed by a detailed survey of the current studies on the successful exfoliation of L‐NvdW 3D‐bonded crystals to obtain thin‐layered 2D materials. The 3D‐bonded crystals considered in this study are categorized into selectively extracted “M” layer and “A” layer of MAX phases respectively, decalcification from layered Zintl phases CaSi*_x_* Ge*_y_* (*x* + *y* = 2), and delocalization of oxides from layered metal oxides. Furthermore, we briefly discuss the applications of these materials and present our perspectives on the challenges and prospects in this field. We anticipate that this manuscript will inspire the synthesis of other novel L‐NvdW nanoplatelets, potentially leading to new applications and advancing our understanding of this intriguing topic.

## 2 General Mechanism and Categories of ECE

**Figure 2:**
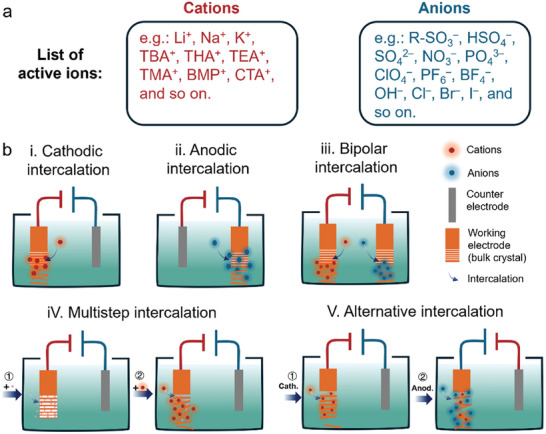
a) List of active ions for electrochemical exfoliation. b) Categories of electrochemical exfoliation: (i) cathodic, (ii) anodic, (iii) bipolar, (iv) multistep, and (v) alternative intercalations.

ECE is an efficient method for producing thin‐layer 2D materials from bulk‐layered parent materials. This process typically employs a standard electrochemical cell with three electrodes: a working electrode (WE), a counter electrode (CE), and, optionally, a reference electrode (RE). The WE is typically used to connect the parent materials to facilitate the accumulation and intercalation of anions and cations in the electrolyte. Only in specific bipolar ECE processes are both the WE and CE made of the parent materials to perform bipolar ECE. As listed in **Figure** **2**a, common cations are alkali ions, quaternary ammoniums, and other organic ions, while typical anions include sulfate, perchlorate, hydroxide, and various halides. The electrolytes used in this process can be either aqueous or nonaqueous solutions, designed to support ionic migration and prevent the restacking of exfoliated sheets.^[^ ^33^, ^35^, ^37^ ^]^

ECE is driven by an external power supply that provides either direct or alternating currents to maintain a constant potential or stable current flow. When a suitable voltage is applied between the working and counter electrodes, ionic species intercalate into the interlayers of the electrode material, causing structural expansion and delamination into thin layers. ECE can be classified into cathodic (Figure 2b‐i) and anodic (Figure 2b‐ii) intercalation based on the applied potential and the intercalated ions. To improve exfoliation efficiency and yield, as well as to produce hybrid materials from the exfoliated 2D nanosheets, various techniques have been developed, including bipolar intercalation (Figure 2b‐iii), multistep intercalation (Figure 2b‐iv), and alternative intercalation (Figure 2b‐v).

Cathodic exfoliation typically involves applying a negative potential, which prompts the positive ions in the solution to intercalate between the layers of the material, causing expansion and exfoliation. Lithium ions are often considered ideal for this purpose due to their small radius, high diffusion coefficient, and the good solubility of lithium salts in nonaqueous solvents, which enhances their intercalation ability.^[^ ^39^, ^40^ ^]^ However, despite their effective intercalation, lithium ions exhibit relatively weak exfoliation capability due to their small size. In contrast, larger organic quaternary ammonium cations, such as TBA^+^ and TMA^+^, demonstrate superior exfoliation performance.^[^ ^41^, ^42^ ^]^ These cations are particularly effective in exfoliating materials with weaker interlayer bonding. Successful examples of such exfoliation include graphite,^[^ ^41^ ^]^ black phosphorus (BP),^[^ ^43^ ^]^ NiPS_3_,^[^ ^44^ ^]^ and CrSBr.^[^ ^45^ ^]^

On the other hand, anodic exfoliation operates by applying a positive potential to drive the intercalation of anions from the electrolyte, or those produced during the electrolysis reaction, into the anodic working electrode in an aqueous solution. Anodic exfoliation is particularly appealing due to its environmentally benign nature, utilizing water or ionic liquids instead of hazardous organic solvents. One of the most notable advancements is the anodic exfoliation of graphite in sulfuric acid and sulfate‐based electrolytes ((NH_4_)_2_SO_4_, Na_2_SO_4_, K_2_SO_4_, etc.).^[^ ^46^, ^47^ ^]^ This method typically offers higher and more rapid production rates compared to other exfoliation techniques. However, unlike cathodic exfoliation, anodically exfoliated graphene materials often exhibit significant oxygen functional group decoration. This functionalization compromises the structural integrity and quality of graphene. These oxygen‐containing groups result from oxidation processes inherent to the application of anodic potentials to the graphite electrode. Addressing this oxidation is critical for improving the quality of anodically exfoliated graphene for advanced applications.^[^ ^48^, ^49^, ^50^ ^]^

Among the numerous 2D materials, some exhibit the capability for both cation and anion intercalation under bias voltage conditions. In these materials, both cations and anions intercalate into the parent material at the respective electrodes, enabling more efficient use of electrical energy and enhancing the exfoliation rate.^[^ ^51^ ^]^ This method has been successfully applied to downsize various materials, including graphite,^[^ ^52^ ^]^ BP,^[^ ^53^ ^]^ transition metal dichalcogenides (TMDs),^[^ ^54^ ^]^ and hexagonal boron nitride (h‐BN).^[^ ^55^ ^]^

In some situations, achieving the desired intercalated structure can be straightforward, involving a single‐step process where a layered host material is directly intercalated. However, the direct intercalation of certain guest species can be challenging due to factors such as size incompatibility or insufficient driving forces. To address these challenges, it is often necessary to employ multiple driving forces or conduct the intercalation through a series of steps.^[^ ^56^ ^]^ As depicted in Figure 2b‐iv, a smaller ion is first pre‐intercalated into the layers to weaken the vdW forces between them. This is followed by the intercalation of a larger ion, which facilitates the exfoliation of the material.

It is worth mentioning that the alternating current power supply can be used in the electrochemical apparatus as schematically depicted in Figure 2b‐v. Feng et al. demonstrated that combining AC power with tetra‐*n*‐butylammonium bisulfate (TBA·HSO_4_) achieves high efficiency and quality.^[^ ^57^ ^]^ O‐containing radicals are generated at graphite/water interfaces on anodes, attacking graphite defects and increasing *d*‐spacing to 0.46 nm. When the potential switches to negative, sulfate anions (HSO_4_^−^) are reduced to gas bubbles, allowing large TBA^+^ cations to intercalate. Conversely, flattened TBA^+^ cations fit between graphene layers in cathodes. As both electrodes expand, complex reactions generate gas bubbles (O_2_, H_2_, SO_2_, CO_2_), resulting in efficient graphene production. The final graphene consists mainly of one to three layers with a high C/O ratio. Wang et al. further improved this method by adding thiourea, which helps control graphite exfoliation.^[^ ^58^ ^]^

## 3 ECE of L‐NvdW Materials

**Figure 3:**
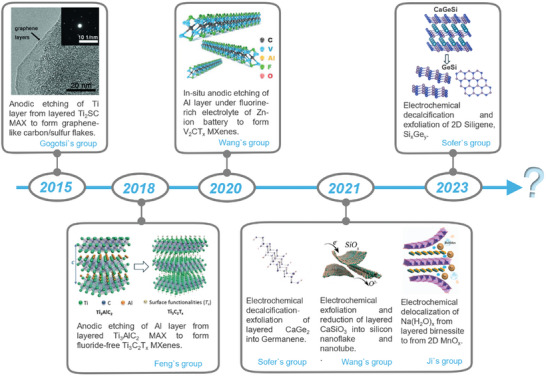
Timeline summarizing the development of electrochemical exfoliation of L‐NvdW 3D‐banded crystal into 2D materials. Reproduced with permission.^[^ ^31^ ^]^ Copyright 2015, Wiley‐VCH. Reproduced with permission.^[^ ^32^ ^]^ Copyright 2018, Wiley‐VCH. Reproduced with permission.^[^ ^65^ ^]^ Copyright 2020, Wiley‐VCH. Reproduced with permission.^[^ ^61^ ^]^ Copyright 2021, American Chemical Society. Reproduced with permission.^[^ ^63^ ^]^ Copyright 2021, American Chemical Society. Reproduced with permission.^[^ ^64^ ^]^ Copyright 2021, Elsevier. Reproduced with permission.^[^ ^62^ ^]^ Copyright 2023, American Chemical Society.

The normal methods to address NvdW 2D materials are chemical vapor deposition (CVD) and solution exfoliations etc. CVD is a bottom‐up method known for producing high‐quality, wafer‐scale films, but it faces challenges such as high‐temperature requirements, complex methodologies, and potential issues during film transfer. In contrast, liquid exfoliation is a more versatile top‐down method that accommodates a broader range of materials but generally yields lower‐quality nanomaterials primarily suited for catalytic applications. Electrochemical exfoliation, while effective only for specific materials, offers tailored nanosheet production and mitigates some drawbacks of both CVD and liquid exfoliation.^[^ ^34^, ^59^, ^60^ ^]^ The exploration of electrochemical exfoliation for obtaining thin‐layered 2D materials from 3D‐bonded L‐NvdW structures has been for nearly a decade. However, this field remains in its early stages, with only intermittent research reports. **Figure** **3** illustrates the development of ECE strategies for L‐NvdW materials and their resulting structures. Key materials of interest include those in the MAX phases, where “M” denotes a transition metal, “A” typically represents Al, Ga, Sn, or S, and “X” stands for C, N, or B. Two notable studies include the work by Gogotsi's group in 2015 on delocalizing the “M” layer in MAX to obtain C/S (“AX” compounds) composites, featuring amorphous carbon and thin‐layer graphene, and the research by Feng's group in 2018 on selectively extracting the A layer from MAX using ECE to produce thin‐layered “MX” compounds, that is, MXene.^[^ ^31^, ^32^ ^]^ Additionally, our group achieved decalcification of layered Zintl phases such as CaGe_2_ and CaSi*_x_* Ge*_y_* in 2021 and 2023, resulting in thin‐layered Germanene and Siligene.^[^ ^61^, ^62^ ^]^ Furthermore, ECE synthesis has been employed to create 2D layered oxides such as MnO_2_ and SiO*_x_*.^[^ ^63^, ^64^ ^]^ The following five subsections provide a comprehensive review of the preparation and development of the aforementioned types of materials.

### 3.1 Delocalization of “A” Layer of L‐NvdW MAX Phases

**Figure 4:**
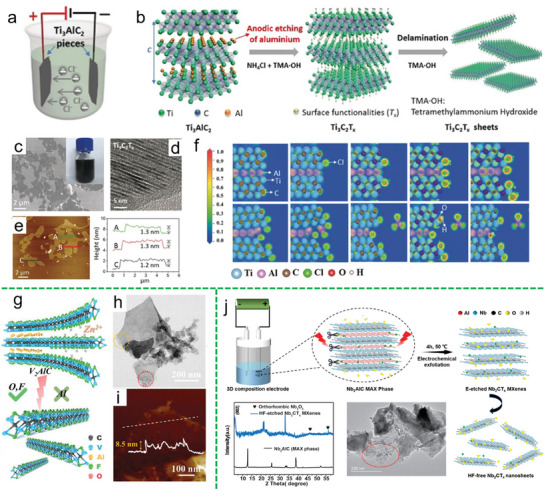
a) Schematic of the electrochemical cell setup used for exfoliation. b) Illustration of the etching and delamination process. c) SEM image, d) HRTEM image, and e) AFM image of the exfoliated Ti_3_C_2_T*_x_*. f) Electron localization function (ELF) plots showing the pristine Ti_3_AlC_2_ and the etched Ti_3_AlC_2_ after the introduction of 1–7 chloride anions. Reproduced with permission.^[^ ^32^ ^]^ Copyright 2018, Wiley‐VCH. g) Diagram illustrating the in situ mechanism of electrochemical etching. h) TEM image and i) AFM image showcasing the exfoliated V_2_CT*_x_*. Reproduced with permission.^[^ ^65^ ^]^ Copyright 2020, Wiley‐VCH. j) Schematic representation of the electrochemical dealuminization process using an HCl‐based electrolyte, along with TEM image and XRD pattern of the resulting F‐free Nb_2_CT*_x_*. Reproduced with permission.^[^ ^68^ ^]^ Copyright 2020, Wiley‐VCH.

Typically, the method for obtaining Ti_3_C_2_T*_x_* MXene from Ti_3_AlC_2_ MAX involves chemically etching the intermediate Al layer using fluoride‐related hazardous chemicals such as HF, LiF/HCl, or NH_4_HF_2_.^[^ ^66^ ^]^ Since chemical etching is fundamentally an electrochemical process driven by electron transfer from aluminum to other substances, electrochemical etching presents a viable alternative.^[^ ^67^ ^]^ In 2018, Feng's group first presented a fluoride‐free electrochemical method for efficiently delaminating Ti_3_C_2_ MXenes in a binary aqueous electrolyte.^[^ ^32^ ^]^ As schematically shown in **Figure** **4**a,b, chloride ions rapidly etch aluminum anodically and break Ti–Al bonds under anodic exfoliation, while ammonium hydroxide (NH_4_OH) helps open the anode edges and facilitates deeper etching. The entire etching process completes in 5 h under ambient conditions, yielding over 90% single or double‐layer materials with lateral sizes above 2 mm, free of fluorine terminations, producing Ti_3_C_2_T*_x_* (T = –O, –OH) flakes (Figure 4c–e). Meanwhile, a density‐functional theory (DFT) calculation was conducted to study the etching mechanism of Ti_3_AlC_2_. As shown in Figure 4f the structure was modelled with Al atoms between two Ti_3_C_2_ layers. Cl⁻ ions were introduced gradually, causing Cl⁻ to attack exposed edges, leading to Al dissociation and AlCl_3_ formation. This process opens grain boundaries, allowing further penetration of NH_4_OH and Cl⁻, which weakens Ti–Al bonds. The electron localization function (ELF) shows strong Ti–C bonds and weaker Ti–Al bonds. Functionalization by OH⁻ significantly weakens Ti‐Al bonds, facilitating Al removal. In addition to Cl⁻‐based electrolytes, F⁻‐based electrolytes also demonstrate excellent performance in electrochemical etching of the Al layer. In 2020, Zhi's group used V_2_AlC MAX as the cathode and Zn foil as the anode, assembling a zinc‐ion button cell with a 21 m LiTFSI + 1 m Zn(OTf)₂ solution as the F‐rich electrolyte for cathodic exfoliation of the MAX phase (Figure 4g–i).^[^ ^65^ ^]^ The V‐Al bonds in the V_2_AlC cathode are broken by the attack of F⁻ ions in the electrolyte, leading to the etching away of Al atoms and the formation of V_2_C. The resulting V_2_C is further oxidized during subsequent cycles to form high‐capacity V_2_O_5_ nanosheets, which can be directly used for Zn‐ion storage. This method is a single‐step, environmentally friendly process that avoids the use of acids or alkalis. The one‐step process confines MAX exfoliation, electrode oxidation, and redox reactions within a sealed cell, preventing any leakage. Notably, the cell continues to function effectively as a rechargeable battery throughout and after exfoliation, with its capacity gradually increasing. Similarly, in 2020, Menglin et al. used 0.5 m HCl as the electrolyte at 50 °C to perform electrochemical dealuminization of Nb_2_AlC, producing F‐free Nb_2_CT*_x_* MXene (Figure 4j).^[^ ^68^ ^]^ All the abovementioned exfoliation strategies rely on the strong selective electrochemical reactions between halide ions and the A layer (e.g., Al) in the MAX phase, effectively removing it while leaving the other components, “M” and “X”. This results in thin, specifically functionalized MXenes.

### 3.2 Delocalization of “M” Layer of L‐NvdW MAX Phases

**Figure 5:**
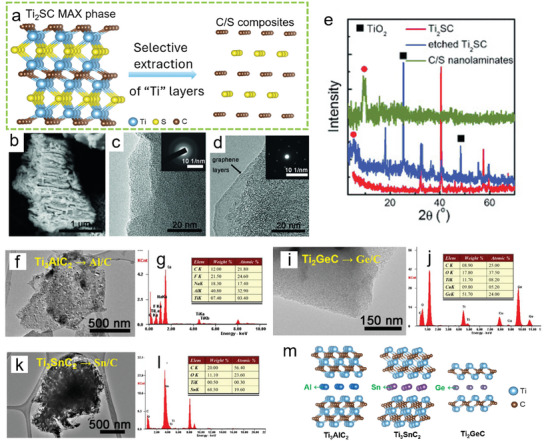
a) Diagram illustrating the selective electrochemical removal of Ti (M) from Ti₂SC (MAX). b) SEM image of a C/S particle. HRTEM images of c) amorphous and d) graphene‐like C/S flakes. e) XRD comparison of Ti_2_SC MAX phases, etched Ti_2_SC and C/S nanolaminates. TEM images with elemental analysis of f,g) Al/C, i,j) Ge/C, and k,l) Sn/C composites. Reproduced with permission.^[^ ^31^ ^]^ Copyright 2015, Wiley‐VCH. m) Crystal structures of Ti_3_AlC_2_, Ti_3_SnC_2_, and Ti_2_GeC.

Interestingly, beyond the selective electrochemical removal of the Al layer in MAX phases to produce “MX” type materials, similar strategies can also be applied to remove the “M” layer elements. This approach enables the formation of “AX” composite materials. As early as 2014, Gogotsi's group conducted cyclic voltammograms on MAX phases in various electrolytes, revealing two distinct anodic steps/peaks. These findings suggested the potential for selectively extracting either the “M” or “A” elements.^[^ ^69^ ^]^ Following this, in 2015, they conducted detailed studies on the selective electrochemical removal of the “M” layer from these materials.^[^ ^31^ ^]^ As shown in **Figure** **5**a, the selective removal of the “M” layer (Ti) from the Ti_2_SC MAX phase was achieved using an F‐based aqueous electrolyte. During the electrochemical etching process, TiO_2_ continuously forms (Figure 5e). Washing the product with HF results in the formation of an S/C composite material. Subsequent TEM images reveal that the carbon in this composite primarily exists in the form of amorphous carbon (Figure 5c) and 2D graphene (Figure 5d). Following, they applied the same strategy to selectively remove Ti from Ti_3_AlC_2_, Ti_2_GeC, and Ti_3_SnC_2_, successfully obtaining Al/C (Figure 5f,g), Ge/C (Figure 5i,j), and Sn/C (Figure 5k,l) composite materials, respectively.

### 3.3 Decalcification of L‐NvdW Zintl Phase CaSi*_x_* Ge*_y_* (*x* + *y* = 2)

Recently, the 2D germanium allotrope, germanene, and its low‐buckled graphene‐like analogs have attracted significant interest due to their unique properties.^[^ ^70^ ^]^ Predicted in 2009, germanene features a narrow bandgap (≈0.024 eV) compared to graphene's zero gap, enabling the quantum spin Hall effect and massless Dirac fermions, making it promising for optoelectronics, sensing, energy storage, and catalysis.^[^ ^71^, ^72^ ^]^ Germanene production has been explored through bottom‐up methods like CVD and MBE, as well as top‐down approaches such as decalcification from CaGe_2_.^[^ ^73^, ^74^ ^]^ For example, Ge‐H was produced by liquid‐phase exfoliation of CaGe_2_ in concentrated HCl, and methyl‐functionalized germanane (Ge‐CH_3_) was synthesized using methyl iodide. The exfoliation of CaGe_2_ can be further expanded to include various alkylation reagents, specifically primary and secondary alkyl iodides.^[^ ^72^, ^75^, ^76^ ^]^ However, these methods typically result in low yields of germanene and involve complex processes that demand harsh acidic conditions and low temperatures.

**Figure 6:**
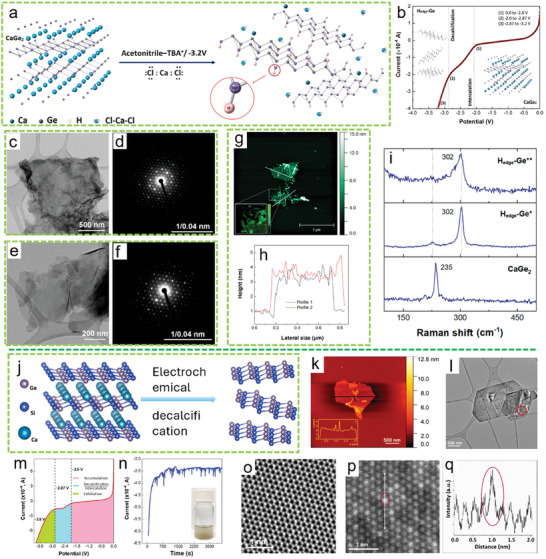
a) Schematic of the electrochemical decalcification mechanism of the CaGe_2_ Zintl phase and b) corresponding voltage polarization curve. c,d) TEM and SAED images of germanane washed only with ACN. e,f) TEM and SAED images of germanane cleaned with acetic acid. g,h) AFM analysis of thin‐layered germanane. i) Raman analysis of CaGe_2_, H_edge_–Ge*, and H_edge_–Ge** samples. Reproduced with permission.^[^ ^61^ ^]^ Copyright 2021, American Chemical Society. j) Schematic of the electrochemical decalcification mechanism of the CaSi*_x_* Ge*_y_* Zintl phase. k,l) AFM and TEM image of prepared siligene. m,n) Electrochemical decalcification curves of CaSi_x_Ge_y_. o) HRTEM image, p) HR‐STEM HAADF image and q) corresponding extracted intensity profile. Reproduced with permission.^[^ ^62^ ^]^ Copyright 2023, American Chemical Society.

In 2021, Kovalska et al. introduced a one‐step method for synthesizing edge‐hydrogenated germanene (H_edge_‐Ge) through ECE of CaGe_2_ in a 0.01 m tetrabutyl ammonium chloride (TBACl) solution in acetonitrile (ACN) (**Figure** **6**a).^[^ ^61^ ^]^ This process involved both intercalation and decalcification occurring simultaneously. Initially, TBA⁺ ions intercalated into the CaGe₂ layers at around −2.2 V, followed by the decalcification step at approximately −2.87 V, which led to the exfoliation of germanene at −3.2 V. The decalcification potential of −2.87 V corresponded to the reduction of Ca^2^⁺ ions (Figure 6b). During the sample purification process, the choice of solvent significantly impacts the final product. Washing with ACN produces H_edge_–Ge* nanosheets, as shown in Figure 6c,d, though these tend to retain surface impurities. A subsequent wash with acetic acid results in cleaner H_edge_–Ge** thin layers, as illustrated in Figure 6e,f. AFM analysis (Figure 6g,h) confirms the successful exfoliation of micrometer‐sized single‐ and few‐layer germanene. Raman spectroscopy (Figure 6i) reveals the formation of a 2D lattice, evidenced by the pronounced E_2__g_ mode at 302.2 cm^−1^ in both H_edge_–Ge* and H_edge_–Ge** samples.

In 2023, Kovalska et al. applied a similar method using 0.03 m tetrabutylammonium perchlorate (TBAClO_4_) in ACN to successfully perform electrochemical decalcification of the CaSi*_x_* Ge*_y_* Zintl phase, resulting in thin layers of siligene (Si*_x_* Ge*_y_*) (Figure 6k,l).^[^ ^62^ ^]^ The procedure was divided into three stages, each with a specific voltage: −2.0, −2.87, and −3.8 V. As shown in Figure 6m,n, the first stage involves the accumulation of tetrabutylammonium cations (TBA⁺) at −2.0 V toward the layered CaSi*_x_* Ge*_y_*. The second stage, at −2.87 V, triggers decalcification and intercalation through the reduction of Ca^2+^ and the diffusion movement of TBA⁺. This potential facilitates the formation of Ca(ClO_4_)_2_ by reducing Ca^2^⁺ and allowing it to interact with ClO_4_⁻ anions in the electrolyte. The final stage, which lasts 5 h at −3.8 V, enables the exfoliation of Si*_x_* Ge*_y_* by completing the decalcification and breaking the bonds in CaSi*_x_* Ge*_y_*. The exceptional crystalline quality of the flake is evident in both the FFT pattern and the HR‐TEM micrograph (Figure 6o). HR‐STEM‐HAADF images (Figure 6p,q) further confirm the crystallinity of the exfoliated siligene and reveal the distribution of Si and Ge atoms within the lattice. In the HAADF images, intensity variations correspond to atomic number and thickness, with the brightest atomic columns identified as Ge atoms. The HR‐STEM‐HAADF images show that Si and Ge atoms are evenly randomly dispersed throughout the lattice, without any signs of agglomeration.

### 3.4 Delocalization of M(H_2_O)*_x_* of M‐Birnessites (M = Li, Na, and K)

**Figure 7:**
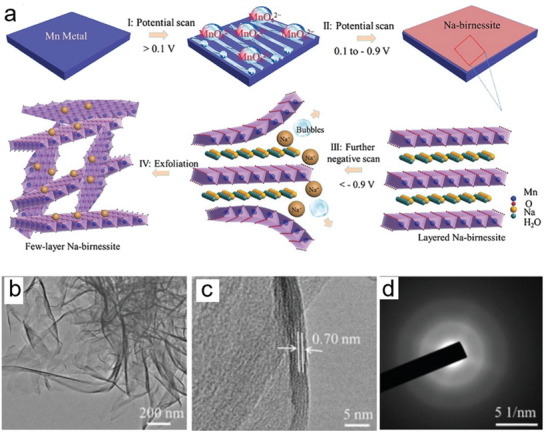
a) Schematic of the ECE mechanism for L‐nvdW bulk Na‐birnessite. b–d) TEM, HRTEM images, and SAED analysis of the prepared 2D birnessite MnO_2_. Reproduced with permission.^[^ ^64^ ^]^ Copyright 2021, Elsevier.

Recent studies have highlighted interesting developments in the ECE of L‐nvdW oxides. In 2021, Yang et al. successfully synthesized a few‐layer 2D birnessite MnO_2_.^[^ ^64^ ^]^ Although birnessite is structured in layers, the interlayer interactions are exceptionally strong, making it a material that lies at the boundary between vdW solids and nvdW solids. The mechanism for ECE of L‐nvdW bulk Na‐birnessite involves several key steps (**Figure** **7**a). First, manganese metal surface atoms oxidize in a strong alkaline solution, forming manganate ions (MnO_4_^2^⁻) when the potential is above 0.1 V (step I). These ions are unstable and quickly reduce to an ultrathin birnessite layer on the metal surface when the potential is reversed between −0.9 and 0.1 V (step II). As the potential shifts below −0.9 V, hydrated cations (e.g., [Na·*n*(H_2_O)]⁺) intercalate into the birnessite layers, leading to gas evolution and layer expansion, causing exfoliation (step III). Repeated potential cycles gradually convert bulk Mn metal into 2D unilaminar or few‐layer birnessite (step IV). This method, using conductive manganese metal, achieves a high exfoliation yield of 96%, overcoming issues of resistance and incomplete exfoliation found in traditional methods. TEM images of prepared 2D birnessite MnO_2_ showed few‐layered sheets with graphene‐like wrinkles and an interplanar spacing of 0.73 nm for the (001) lattice planes (Figure 7b,c). The SAED patterns in Figure 7d display two prominent diffraction rings, which correspond to the (0 0 1) and (0 0 2) crystalline planes characteristic of birnessite.

### 3.5 Delocalization of CaO*_x_* of L‐NvdW Silicate (CaSiO_3_)

**Figure 8:**
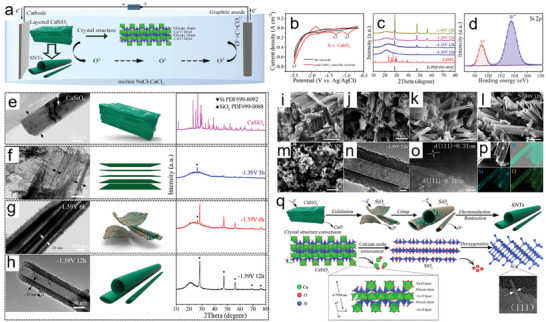
a) Diagram illustrating the formation process of SNTs during electrolysis. b) CV curves comparing Mo and CaSiO_3_‐modified Mo electrodes at 50 mV s^−1^ in molten NaCl–CaCl_2_ at 800 °C. c) XRD patterns showing the phase evolution of electrolytic products under different potentials. d) High‐resolution XPS spectra of Si 2p for SNTs obtained at −1.39 V after 12 h. e) TEM images and XRD patterns of the original CaSiO_3_ material. f–h) TEM images and XRD patterns of CaSiO_3_ after electrolysis at −1.39 V for 3, 6, and 12 h. i) SEM images of pristine CaSiO_3_. j–m) SEM images of products following electrolysis at −1.28, −1.35, −1.39, and −1.48 V for 12 h. n–p) TEM images of SNTs synthesized at −1.39 V for 12 h. q) Schematic representation of the SNT formation mechanism during electrolysis. Reproduced with permission.^[^ ^63^ ^]^ Copyright 2021, American Chemical Society.

A noteworthy study on the ECE of L‐NvdW oxides is the 2021 work by Wang et al.^[^ ^63^ ^]^ By electrochemically removing the CaO_x_ interlayer from CaSiO_3_ in a NaCl–CaCl_2_ molten salt, SiO*_x_* nanosheets can be produced. Under exfoliation stress, these nanosheets tend to curl, forming SiO*_x_* nanotubes. Further electrochemical reduction of these nanotubes leads to the formation of thin silicon nanotubes (SNTs). The process for electrochemically synthesizing SNTs is illustrated in **Figure** **8**a,q. To expedite the analysis of this electrochemical reduction, CaSiO_3_ powder was placed in a Mo cavity, with graphite serving as the counter electrode and Ag/AgCl as the reference electrode. Figure 8b shows CV curves recorded at 50 mV s^−1^ in a 50:50 mol% NaCl–CaCl_2_ molten salt at 800 °C, comparing a CaSiO_3_‐loaded Mo electrode with a blank Mo electrode. The CaSiO_3_ electrode displays two reduction peaks at −0.97 and −1.3 V (vs Ag/AgCl), corresponding to the stepwise reduction of CaSiO_3_ to elemental Si, indicating a multistage conversion during the electrochemical reduction. Figure 8c,d further confirms the formation of Si through XRD and XPS analyses conducted on the materials during synthesis. Additionally, comprehensive ex situ TEM, XRD, and SEM analyses shown in Figure 8e–p provide a detailed account of the material's transformation from a layered CaSiO_3_ 3D bulk structure to expanded CaSiO_3_ sheets, followed by the formation of SiO*_x_* nanosheets after delocalization of CaO*_x_*, and eventually, the generation of SiO*_x_* nanotubes and SNTs during the subsequent reduction process of losing O^2−^.

## 4 Application of Materials obtained from L‐NvdW Compounds

Currently, research on 2D materials, particularly vdW 2D materials, is the most extensive. In contrast, studies on NvdW structured materials typically focus on those that retain the same composition as the parent material after exfoliation.^[^ ^33^ ^]^ Particularly, there are only a few reports on the selective delocalization of specific components from parent L‐NvdW materials via electrochemical methods to obtain thin‐layer 2D materials, and this is also reflected in the limited research on their applications. In this section, we review all current applications of materials obtained from ECE of L‐NvdW compounds, including energy storage, electronic devices, and sensors.

### 4.1 Energy Storage

2D materials have gained significant interest as electrodes in energy storage devices, such as batteries and supercapacitors, due to their open structure, tunable charge transport, and superior redox properties. These features enhance diffusion, energy carrier interactions, and mechanical stability, leading to improved charge transport and storage capacity.^[^ ^3^, ^77^ ^]^

**Figure 9:**
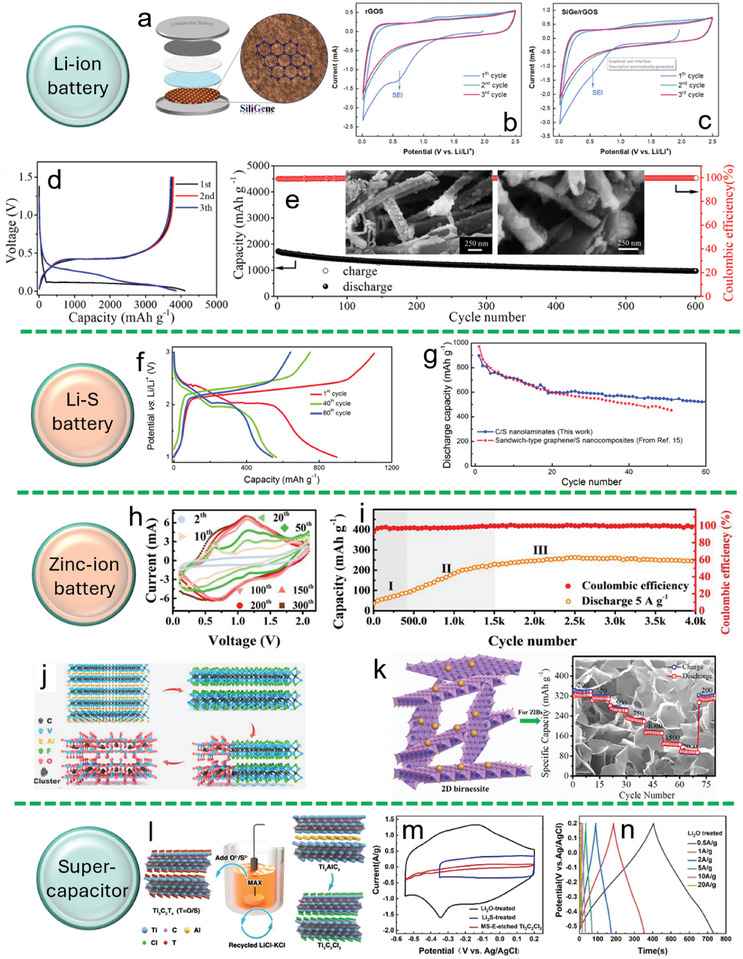
Li‐ion battery applications: a) Schematic of a lithium‐ion battery. b) CV curve of rGO. c) CV curve of rGO/siligene composite. Reproduced with permission.^[^ ^62^ ^]^ Copyright 2023, American Chemical Society. d,e) Charge–discharge profiles and long‐term cycling performance of SNTs; inset shows SEM images before and after cycling. Reproduced with permission.^[^ ^63^ ^]^ Copyright 2021, American Chemical Society. Li–S battery: f,g) Charge–discharge profiles and cycling life of C/S in a Li–S battery system. Reproduced with permission.^[^ ^31^ ^]^ Copyright 2015, Wiley‐VCH. Zinc‐ion battery: h) In situ CV activation of V_2_AlC MAX. i) Cycling stability of the ZIB. j) Schematic of V_2_AlC transformation into V_2_CT*_x_* and V_2_O_5_. Reproduced with permission.^[^ ^65^ ^]^ Copyright 2020, Wiley‐VCH. k) Rate performance of 2D Na‐birnessite. Reproduced with permission.^[^ ^64^ ^]^ Copyright 2021, Elsevier. Supercapacitor: l) Molten‐salt electrochemical etching for F‐free Ti_3_C_2_Cl*_x_*. m,n) CV and GCD curves at various current densities. Reproduced with permission.^[^ ^80^ ^]^ Copyright 2021, Wiley‐VCH.

The solid‐electrolyte interphase (SEI) is crucial for lithium and sodium batteries, though excessive SEI formation can lead to capacity loss and obstruction of Li^+^ transport. In 2023, Kovalska et al. demonstrated that siligene obtained via ECE can regulate SEI formation on rGO, thereby enhancing both initial Coulombic efficiency and cycling stability in lithium‐ion batteries (**Figure** **9**a–c).^[^ ^62^ ^]^ In 2021, Wang et al. utilized Si nanotubes derived from CaSiO_3_ in lithium‐ion batteries, where the unique hollow structure resulted in superior Li‐storage performance, including high capacity and stability (Figure 9d,e).^[^ ^63^ ^]^

In 2015, Gogotsi et al. developed a method to extract Ti from Ti_2_SC laminates and TiO_2_, leading to the creation of C/S nanolaminates.^[^ ^31^ ^]^ These nanolaminates, tested as cathodes in Li–S batteries, showed strong electrochemical performance, with initial discharge capacities around 900 mAh g⁻^1^, which decreased to about 530 mAh g⁻^1^ after 60 cycles. The C/S nanolaminates also demonstrated better cycling stability compared to graphene/S composites under similar conditions (Figure 9f,g). The potential of this unique structure in Li–S batteries was further validated by Hasegawa et al.^[^ ^78^ ^]^

In 2020, Zhi et al. demonstrated a novel approach by using a specialized F‐rich electrolyte to directly exfoliate MAX V_2_AlC within a battery, leading to the in situ formation of V_2_CT*_x_* MXene.^[^ ^65^ ^]^ This one‐step process is confined entirely within the cell, effectively eliminating the risk of external contamination. Over its operational life, the battery experiences three critical stages: exfoliation, oxidation of the electrode, and redox reactions involving V_2_O_5_ (Figure 9j). Remarkably, despite these ongoing changes, the battery continues to operate efficiently, with performance consistently improving (Figure 9h,i). The resulting aqueous zinc‐ion battery exhibits exceptional durability, maintaining stability over 4000 cycles, and demonstrates impressive rate performance of 97.5 mAh g⁻^1^ at 64 A g⁻^1^. This performance not only surpasses that of all previously reported aqueous MXene‐based batteries but also exceeds the capacity of most vanadium‐based zinc‐ion batteries.^[^ ^12^, ^79^ ^]^

In 2021, Ji's group developed a one‐pot electrochemical potential cycling method h to exfoliate nonconductive 2D layered birnessite‐type manganese oxide from a non‐layered metal precursor.^[^ ^64^ ^]^ By adjusting the potential sweep rate, monolayer and few‐layer birnessite sheets were successfully produced. When used as a cathode material for Zn‐ion batteries, the exfoliated birnessite exhibited excellent rate performance, achieving a capacity of 325 mAh g⁻^1^ at 2000 mA g⁻^1^ (Figure 9k).

Ti_3_C_2_T*_x_* is widely recognized for its excellent performance in supercapacitors due to its pseudocapacitive storage mechanism. In 2021, Wang's group fabricated a symmetric supercapacitor using F‐free Ti_3_C_2_Cl_x_ MXene, prepared via molten‐salt assisted electrochemical exfoliation (Figure 9l). Furthermore, surface terminations can be in situ modified from −Cl to −O and/or −S, significantly shortening modification steps and increasing surface termination diversity. The resulting −O‐terminated Ti_3_C_2_T*_x_* serves as excellent electrode material for supercapacitors, showing capacitances of 225 F g^−1^ at 1.0 A g^−1^ and good rate capability of 91.1 % at 10 A g^−1^ (Figure 9m,n).

### 4.2 Electronic Devices

**Figure 10:**
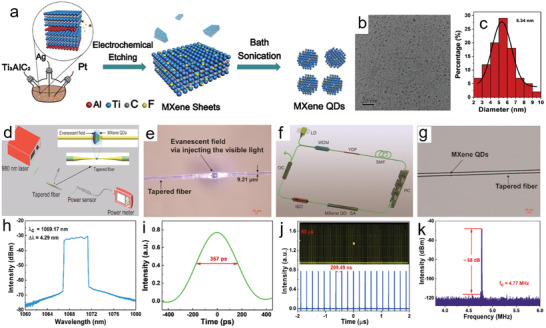
a) Schematic of the setup for synthesizing Ti_3_C_2_T*_x_* QDs. b,c) TEM image and size distribution of Ti_3_C_2_T*_x_* QDs. d–g) Diagram of the photodeposition process, showing light leakage in the waist region, the laser setup, and the Ti_3_C_2_T*_x_* QD saturable absorber after deposition. h–k) Optical spectrum of dissipative solitons, single pulse profile, pulse trains over 50 and 4 µs, and the RF spectrum at the fundamental frequency. Reproduced with permission.^[^ ^81^ ^]^ Copyright 2020, American Chemical Society.

In 2020, Yang et al. synthesized MXene quantum dots (QDs) using a three‐electrode electrochemical cell (**Figure** **10**a). The process lasted 5 h, during which PF_6_^−^ decomposed into F^−^, selectively etching the Al layer from Ti_3_AlC_2_. The resulting brown suspension was centrifuged twice—first at 3500 rpm and then at 7000 rpm—to separate large particles. The sediment was then sonicated in MeCN under N_2_ for 10 h and centrifuged again to obtain the Ti_3_AlC_2_ MXene QDs in the supernatant. Figure 10b,c presents the TEM image of the QDs and their size distribution, with an average lateral size of ≈5.34 nm. A fluorinated Ti_3_C_2_T*_x_* QD dispersion was deposited onto a microfiber using a 980 nm laser, as shown in Figure 10d–g. The microfiber, created via the flame taper method (waist diameter: ≈9.21 µm; insertion loss: 0.69 dB), was placed in a cavity (Figure 10f). When the laser power reached 25 mW, light leaked through the tapered fiber's evanescent field, trapping the QDs in the waist region (Figure 10e). The device had an interaction length of ≈200 µm, with real‐time power measurements monitored at the fiber's opposite end. The optical spectrum (Figure 10h ) showed sharp spectral edges, typical of dissipative solitons in YDFL fiber lasers, centered at 1069.17 nm with a 3 dB bandwidth of 4.29 nm. The pulse width (FWHM) was 357 ps (Figure 10i), yielding a time‐bandwidth product of ≈401.93, indicating heavy chirping, a characteristic of dissipative solitons. The uniform pulse train (Figure 10j) had a time interval of 209.49 ns, matching the cavity length, with no noticeable power jitter, indicating stable mode‐locking. The signal‐to‐noise ratio (SNR) reached ≈68 dB at a fundamental repetition rate of 4.77 MHz (Figure 10k).

### 4.3 Sensors

**Figure 11:**
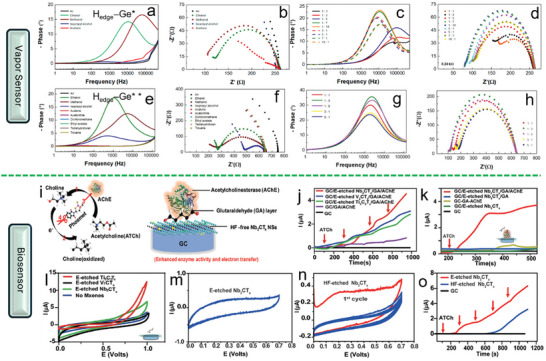
Vapor sensor characterization: a,c) Bode and b,d) Nyquist diagrams for H_edge_–Ge*‐based vapor sensors, and e,g) Bode and f,h) Nyquist diagrams for H_edge_–Ge**‐based vapor sensors. Reproduced with permission. Reproduced with permission.^[^ ^61^ ^]^ Copyright 2021, American Chemical Society. Biosensor Performance: i) Enzyme inhibition effect for phosmet detection using an HF‐free Nb_2_CT*_x_*/AChE biosensor. j) Chronoamperometric response of HF‐free MXene‐modified biosensors with successive ATCh injections. k) Chronoamperometric measurements on different layers after ATCh injection. l) Electrocatalytic activity of HF‐free MXene with acetylcholine at 50 mV s⁻.^1^ m) CV of CE/E‐etched Nb_2_CT*_x_* and n) CE/HF‐etched Nb_2_CT*_x_* showing 1st to 5th CV scans from 0 to +0.7 V at 10 mV s⁻^1^. o) Performance comparison of HF‐etched versus E‐etched Nb_2_CT*_x_* biosensors. Reproduced with permission.^[^ ^68^ ^]^ Copyright 2020, Wiley‐VCH.

In 2021, Kovalska et al. tried two kinds of edge‐hydrogenated germanene, H_edge_–Ge* (**Figure** **11**a–d) and H_edge_–Ge** (Figure 11e–g), for vapor sensors.^[^ ^61^ ^]^ The sensors, based on H_edge_–Ge* and H_edge_–Ge**, showed initial resistivities of 0.24 (Figure 11a) and 0.80 kΩ (Figure 11e), respectively, and remained stable after multiple tests. Both sensors demonstrated sensitivity to ethanol (EtOH), methanol (MeOH), isopropyl alcohol (IPA), and acetone, with notable increases in resistivity when exposed to these VOCs, particularly MeOH and EtOH. The sensitivity was further analyzed through Bode and Nyquist diagrams (Figure 11b–d,f–h), showing frequency and phase shifts upon VOC adsorption. The sensors exhibited higher sensitivity to MeOH over EtOH, likely due to the stronger interaction between the germanene surface and the more symmetric, less polar MeOH. Selectivity tests with varying ratios of EtOH and MeOH revealed that the sensors' peak frequency shifted according to the dominant compound, with resistance increasing in MeOH and decreasing in EtOH, confirming their selective sensitivity.

Song et al., utilized 2D fluoride‐free Nb_2_CT*_x_* MXene nanosheets through an ECE route for an enzymatic electrochemical biosensor. As shown in Figure 11i, the inhibition effect of phosmet on this sensor highlights its high sensitivity and stability. The biosensor, fabricated by coating a glassy carbon electrode (GCE) with glutaraldehyde and immobilizing AChE, demonstrated strong electrochemical reversibility (Figure 11j) and a significant current response after AChE immobilization (Figure 11k). Notably, the ECE‐obtained Nb_2_CT*_x_* sensor maintained stable performance with minimal current decrease over multiple cycles, unlike the HF‐etched counterpart, which showed a significant drop due to NbO*_x_* formation (Figure 11l–n). Ultimately, the ECE‐obtained Nb_2_CT*_x_*‐based biosensor exhibited much higher sensitivity for OP detection, as demonstrated in Figure 11o, making it a robust and promising platform for electrochemical sensing.

## 5 Conclusion and Perspectives

In this review, we have explored the emerging field of electrochemical exfoliation of 3D‐bonded L‐NvdW crystals. After briefly explaining the fundamental principles and classifications of various ECE methods, we surveyed the development of representative NvdW materials over the past decade, focusing on key advancements in their preparation. We provided a detailed summary of all reported materials (**Table** **1**) and methods related to this topic, categorized into five distinct groups based on their structure and the components delocalized from the parent crystal: (1) delocalization of the “M” layer in MAX phases; (2) delocalization of the “A” layer in MAX phases; (3) decalcification of Zintl phase CaSi*_x_* Ge*_y_*; (4) delocalization of M(H_2_O)*_x_* in M‐birnessite (M = Li, Na, K); and (5) delocalization of CaO*_x_* in L‐NvdW silicate (CaSiO_3_). Finally, we discussed the potential applications of these materials and drew connections between the results from different materials to gain deeper insights into this rapidly evolving field.

**Table 1:** A summary of electrochemical delamination to prepare 2D nanosheets from L‐NvdW materials.

2D materials	Starting materials	Delocalized elements	Methods	Electrolytes	Active ions	Thickness/layers	Refs. & Application
S/C nanolaminates	Ti_2_SC	Ti	Anodic, 0–2 V vs Ag/AgCl	0.5 m NH_4_F, 20% HCl, or 5% HF aqueous solution	F^−^	thin‐layered graphene flakes	^[^ ^31^ ^]^ Li–S battery
Al/C nanolaminates	Ti_3_AlC_2_	Ti	Anodic, 0–2 V vs Ag/AgCl	0.5 m NH_4_F in H_2_O	F^−^	thin‐layered flakes	^[^ ^31^ ^]^ –
Ge/C nanolaminates	Ti_2_AlC	Ti	Anodic, 0–2 V vs Ag/AgCl	0.5 m NH_4_F in H_2_O	F^−^	thin‐layered flakes	^[^ ^31^ ^]^ –
Sn/C nanolaminates	Ti_3_SnC_2_	Ti	Anodic, 0–2 V vs Ag/AgCl	0.5 m NH_4_F in H_2_O	F^−^	thin‐layered flakes	^[^ ^31^ ^]^ –
Ti_3_C_2_T*_x_*	Ti_3_AlC_2_	Al	Anodic, +5 V, min	1 m NH_4_Cl+0.2 m TMAOH in H_2_O	Cl^−^	1.3 nm, monolayer	^[^ ^32^ ^]^ Capacitor
S/C compound	Ti_2_SC	Ti	Anodic, 0.8 V vs Ag/AgCl	0.5 m NH4F in H_2_O	F^–^	amorphous S/C compound	^[^ ^78^ ^]^ Li–S battery
V_2_CT*_x_*	V_2_AlC	Al	Battery cycling, 0–2 V vs Zn/Zn^+^	0.21 m LiTFSI^+^ 0.01 m Zn(OTF)_2_ in H_2_O	Zn^+^ and F^−^	8.5 nm, V_2_CTX nanoflakes	^[^ ^65^ ^]^ Zinc‐ion battery
Nb_2_CT*_x_*	Nb_2_AlC	Al	Anodic, 1 V	0.5 m HCl at 50 °C	Cl^−^	Thin‐layered flakes	^[^ ^68^ ^]^ Biosensor
Ti_3_C_2_T*_x_*	Ti_3_AlC_2_	Al	Anodic, 3–7 V vs Ag/Ag+	20% (m/v) [EMIM][PF_6_] in MeCN	PF_6_^−^,F^−^	thin‐layered flakes	^[^ ^81^ ^]^ Photonics
Germanene	CaGe_2_	Ca	Cathodic, −3.2 V	0.01 m TBACl in ACN	TBA+, Cl^−^	≈2 nm, bilayer	^[^ ^61^ ^]^ Vapor sensor
SiO*_x_* nanoflake, rolled SiO*_x_* and Si nanotubes	CaSiO_3_	CaO*_x_*	Cathodic, −1.28 V to −1.48 V vs Ag/AgCl	NaCl:CaCl_2_ = 1:1 molten salt at 800 °C	–	Nanoflakes and nanotubes	^[^ ^63^ ^]^ Li‐ion battery
MnO_2_	M*_x_* MnO_2_⋅*y*H_2_O, (M = Li, Na, K)	M*_x_* ⋅*y*H_2_O	CV scanning, 2 to −5.0 V vs Hg/HgO	10 m MOH in H_2_O (M = Li, Na, K)	Li^+^, Na^+^, or K^+^	Thin‐layered flakes	^[^ ^64^ ^]^ Zinc‐ion battery
Ti_3_C_2_F*_x_*	Ti_3_AlC_2_	Al	Anodic, 3–7 V vs Ag/Ag+	10 m [BMIM][PF_6_] in MeCN	PF_6_^−^,F^−^	Thin‐layered flakes	^[^ ^82^ ^]^ Li‐ion battery
SiliGene	CaSiGe	Ca	Cathodic, −3.8 V	0.03 m TBAClO_4_ in ACN	TBA^+^, ClO_4_^−^	≈2 nm, bilayer	^[^ ^62^ ^]^ Li‐ion battery
Ti_3_C_2_Cl_2_	Ti_3_AlC_2_	Al	Anodic, 1.204 V vs Ag/AgCl	LiCl+KCl molten salt at 450 °C	Cl^−^	Thin‐layered flakes	^[^ ^80^ ^]^ Capacitor

### 5.1 Understanding Exfoliation Mechanisms

Over the past decade, significant progress has been made in exfoliating L‐vdW crystals using ECE methods, resulting in a diverse range of materials and applications. In contrast, the electrochemical exfoliation of L‐NvdW crystals is still in its early stages. Despite this, the field holds great promise, with the potential to yield novel 2D nanosheets with unique properties. However, several challenges and critical questions need to be addressed before this field can fully mature. One of the most important aspects probably is understanding the exfoliation mechanisms of L‐NvdW materials and the relationship between experimental conditions and the inherent properties of these materials: (1) For example, Gogotsi used an F‐based aqueous electrolyte to selectively remove the “M” component (Ti) from Ti_3_AlC_2_ MAX, obtaining an Al/C composite. In contrast, when Yang et al. switched the electrolyte to [EMIM][PF_6_] in MeCN, they selectively removed the “A” component (Al), producing Ti_3_C_2_T*_x_* MXene. This suggests that the electrolyte composition influences the electrochemical reactivity of elements in the parent material. (2) Additionally, the anodic method used for delocalizing “M” or “A” from MAX phases involves halide anions (Cl⁻/F⁻) reacting with the respective elements. However, in the Zintl phase CaSi*_x_* Ge*_y_*, which has a structure similar to MAX phases, the cathodic method is employed, where cations (e.g., TBA^+^) disrupt Ca–Si/Ge bonds, and the Ca element is subsequently removed. In contrast, the exfoliation of layered M‐birnessite, where M has a moderate binding force with MnO_2_ layers, can be successfully achieved using smaller alkali metal cations through cathodic exfoliation. CaSiO_3_ is unique in that it undergoes electrochemical reduction in a high‐temperature NaCl−CaCl_2_ molten salt environment, converting from CaSiO_3_ to SiO*_x_* to Si. (3) Moreover, some L‐NvdW materials acquire new surface terminations after delocalization, but the exact composition and microstructure of these groups remain difficult to determine.

### 5.2 Methodological Diversity

Beyond these challenges, several other aspects warrant further investigation: While ECE methods for L‐vdW crystals have advanced significantly, employing cathodic, anodic, bipolar, multistep, and alternative intercalations, current studies on L‐NvdW exfoliation have only utilized cathodic or anodic methods. Exploring different techniques could improve exfoliation efficiency, surface structure control, and the development of new materials.

### 5.3 Broadened Material Exploration

ECE has only been successfully applied to a limited set of L‐NvdW materials listed in Table 1. Even within the MAX phase family alone, 342 different structures have been cataloged, providing ample opportunity for further exploration.^[^ ^83^ ^]^ Additionally, other birnessite variants, such as Ca‐ and Ba‐based birnessite, share similar layered structures with Na‐birnessite, making them intriguing subjects for future research.

### 5.4 Tailored Electrolyte Selection

Manipulating the electrolyte composition could lead to the discovery of new materials. For example, while ECE in MAX phases has so far only been reported with F⁻ and Cl⁻ electrolytes, exploring other halides like Br⁻ and I⁻ could yield new insights. However, different material systems have distinct electrolyte requirements for dissolution. MAX and Zintl phases rely on anions like F⁻ and Cl⁻ to dissolve specific metallic layers while preserving others. In contrast, M‐birnessite materials utilize alkali metal cation intercalation to weaken interlayer bonding, expelling associated components. Silicates, such as CaSiO_3_, were exfoliated in high‐temperature molten salt environments through the self‐reduction of silicon and the melting of interlayer CaO, without the need for ion intercalation. These findings highlight the necessity of tailored electrolyte selection for each material system.

### 5.5 Postprocessing Opportunities

Post‐processing of these materials, such as defect engineering and compositional tuning, offers further research opportunities. For instance, in this review, Ti_2_SC was used to prepare S/C composites, and the presence of TiO_2_ as a byproduct may enhance the adsorption and catalytic conversion of Li*_x_* S during battery reactions, improving performance.

### 5.6 Adjusting Reaction Parameters

Adjusting parameters such as current density and response time during the electrochemical exfoliation process can significantly influence the efficiency of exfoliation as well as the size and structural integrity of the resulting 2D nanosheets. Specifically: (1) By varying the current density, we can control the rate of ion intercalation and exfoliation, which directly affects the thickness and size of the produced flakes. Higher currents may lead to faster exfoliation but could also introduce more defects due to rapid material removal. (2) Prolonging the electrochemical reaction time allows for more complete exfoliation; however, it may also increase the risk of inducing structural defects or altering the properties of the nanosheets. This control over the exfoliation process is crucial for tailoring the properties of the resulting materials for specific applications.

Finally, as research on these materials is still in its infancy, their practical applications remain largely unexplored, presenting a broad area for future studies.

## Conflict of Interest

The authors declare no conflict of interest.
